# The Effect of Cognitive Behavioral Therapy on Marital Quality among Women 

**DOI:** 10.22074/ijfs.2018.5257

**Published:** 2018-03-18

**Authors:** Arezoo Shayan, Masumeh Taravati, Maryam Garousian, Narges Babakhani, Javad Faradmal, Seyedeh Zahra Masoumi

**Affiliations:** 1 School of Nursing and Midwifery, Chronic Diseases (Home Care) Research Center, Hamadan University of Medical Sciences, Hamadan, Iran; 2 School of Nursing and Midwifery, Hamadan University of Medical Sciences, Hamadan, Iran; 3 Fatemieh Hospital, Hamadan University of Medical Sciences, Hamadan, Iran; 4 Department of Biostatistics, School of Public Health, Hamadan University of Medical Sciences, Hamadan, Iran

**Keywords:** Cognitive Behavioral, Counseling, Marital Relationship

## Abstract

**Background:**

Marital quality reflects the individual’s overall evaluation of marital relationship. The aim of study was examine the effect of cognitive behavioral counseling on marital quality among women.

**Materials and Methods:**

The experimental study was a randomized clinical trial with two groups, on 198 qualified women who referred to selected health care centers in Hamadan, Iran in 2016. The intervention participants attended four 90-minute sessions of cognitive behavioral counseling. Demographic information questionnaire and marital qual- ity scale [Revised Dyadic Adjustment Scale (RDAS)] were completed by the two groups before and after the interven- tion. To perform the comparisons, t test, Chi-square test and Fisher’s test, Logistic Regression and covariance analysis were used. Covariance analysis or change analysis were employed. Statistical analysis was done using SPSS Software, version 21.0. The significance level was set at 5% (P<0.05).

**Results:**

According to the results of the present study, the mean age in the control group and the intervention group was 23.58 ± 7.54 and 35.04 ± 7.91 years old, respectively. Covariance analysis was utilized to examine the marital quality scores. In this analysis, after modification of the variables of age, marital quality score of agreement and sat- isfaction before the intervention, and income status, the total marital quality score experienced a significant change in all dimensions (P<0.05) and the mean scores increased remarkably. Moreover, according to the cut-off point of the dimensions, the scores of all dimensions increased remarkably and the proportion of individuals with high marital quality before and after the intervention changed significantly (P<0.05).

**Conclusion:**

Due to the role of sexual relations in stabilizing marriage, cognitive behavioral consultation was effective in improving marital quality especially after agreement and can be used in health care centers in order to improve the relationship between couples and reduce divorce rates (Registration number: IRCT201610209014N125).

## Introduction

Marital quality has been studied in positive psychology and its role in interpersonal relationships, especially in marital relationships, is remarkable. According to Marx, “quality of marital relations is the result of methods through which married individuals systematically organize this triangle”. The quality of marital relations and the level of happiness depend on how wife and husband interact with each other and cope with stressful situations of life (1). 

Marital quality reflects the individual’s total evaluation of the marital relation (2). The third approach is Marx’s theory which is a combined approach developed based on Lewis and Spanier approach and Bowen systematic approach. Marx has a systematic attitude towards individual and the individual’s relationship with spouse and others. In his theoretical framework, he stated that a married person has three angles including inner angel, spouse angle, and effective person. 

The first angel is the inner angel that includes the individual’s personality with his efforts, motivations, and different energies which is formed based on his experience during life. The second angle is the relationship with the spouse. The third angle is any outside concentration point except for the spouse. Generally, Marx believes that marital quality is the result of the methods by which married individuals systematically organize themselves within this triangle (2, 3).

Sex education is one of the factors that improves the relationship between couples and enhances marital quality. Lack of enough information about sex and improper attitudes towards this issue in families and couples are among the major problems that today’s Iranian society is facing, and leads to collapse of the family. In this regard, marital counseling as a specialized consultation can convey the information necessary to create a favorable sexual life to the couples, so that they can utilize this information to evolve and complete their marriage. In this regard, as midwives are in constant contact with the community and due to their awareness about sexual issues , they play a significant role in making sure about the couples’ satisfaction with their marital life, whereby a step towards creating a healthy society, will be taken (4, 5). Statistics indicates that 50% of couples experience sexual dysfunction in some stages of their lives; however, few undergo consultation or treatment (6). 

By providing sex education and consultation, sexual problems could be diminished gradually, and unawareness will be replaced with complete awareness. Marital counseling for prevention of marital difficulties is one of the most effective methods of development of individuals and couples health education. 

Sexual counseling plays a significant role in family health, as it can decrease sexual violence in the family, prevent sexually transmitted diseases, result in a positive attitude towards sexual relations and sexual pleasure, decrease conflicts within the family, and gain pleasurable sexual experience and sexual satisfaction. Consultation is a process that helps to improve sexual health, interpersonal relationships, affection, intimacy, body image, and gender roles. Sexual counseling is related to a cognitive domain (information and knowledge), affection domain (feelings, values, and attitudes), and behavioral domain (communication skills and decision making) (7).

Cognitive behavioral therapy is today’s most popular and widely-used model of psychotherapy, and clinical studies have proven its efficiency in different populations and for treatment of various problems. This approach is characterized by short-term and problem-focused cognitive behavioral intervention strategies that are retrieved from science and cognitive and learning theories (8, 9). In cognitive behavioral approach, an individual learns to fight against his/her negative attitudes toward sexual issues and improve his/her interpersonal relationships by utilizing his/her problem-solving ability. Moreover, this approach helps to promote and maintain good physical and mental feelings between the couples (10).

Cognitive behavioral consultation approach is one of the most common methods of treating sexual dysfunctions (11). The term “cognitive behavioral consultation” is used to refer to an approach in which it is necessary to cope with overt and cognitive components of behavior. Although traditional behavior therapies are still uniquely important, it is believed that intervention affects cognitive aspects of behavior. Most programs that are designed to treat sexual dysfunctions also use behavioral approaches and are based on this premise that cognitive change cause behavioral changes as well (12). 

This approach was created by combining behavior therapy approach and cognitive approach either in the form of cognitive therapy or the framework of cognitive psychology and basic cognitive science. In cognitive behavioral therapy, strengths of behavior therapy and cognitive therapy, i.e. objectivism, evaluation, and assessment on one hand and the role of memory in reconstructing and interpreting data, on the other hand, are collected and become one entity. Nowadays, this approach involves relatively different theories and attitudes. Unlike other forms of behavior therapies, cognitive behavioral methods directly deal with thoughts and feelings that are obviously significant in all psychological disorders. Cognitive behavioral therapy fills the gap felt by most merely-behavioral methods and dynamic psychotherapy (13).

One of the main components of this therapy is presenting sexual knowledge and information related to sexual response cycle, anatomy, and sexual techniques (14).

Therefore, due to the importance of sexual relationships and their effect on family and society health, significance of presentation of educational and counseling programs in healthcare centers, and limited studies conducted to confirm the effectiveness of this consultation on marital quality of individuals, the present study was done to examine the effect of cognitive behavioral consultation on marital quality among women referring to healthcare centers in Hamadan, Iran.

## Materials and Methods

The experimental study was carried out as a randomized clinical trial including an intervention group (n=99) and a control group (n=99) with a pretest and a posttest on qualified women referring to selected health care centers of Hamadan. The sample size was calculated using formula, $n=\frac{{\left(Z_{1-\frac{\alpha }{2}},+,Z_{1-\beta }\right)}^{2}\left(S_{1}^{2},+,S_{2}^{2}\right)}{{\left(d\right)}^{2}}$ where S is standard deviation and indices 1 and 2 referring to the intervention and control groups were both equal to 16.1 according to reference (15), d was equal to 10 and Z indicated percentile of normal distribution which was calculated for indices 0.975 and 0.80. Based on this information, the sample size was determined to be 82 in each group, and the final sample size was calculated as 198 considering a 20% loss.

This research plan with codes of ethics was accepted by the Chronic Diseases Research Center, Hamadan University of Medical Sciences, Hamadan, Iran (IR.UMSHA.REC.1394.573) and (IRCT201610209014N125).

## Study inclusion criteria

Married women of 15-45 years old (reproductive age), Literacy, Having >6 months of married life, Hamadan residency, No background of remarkable physical and psychological diseases such as psychotic disorders like schizophrenia and severe depression that need special medicine or diet and filling out the questionnaires, age difference of 10 years between the couples.

## Study exclusion criteria

Becoming pregnant during the study, Unwillingness to continue the trial, addiction to drugs and/or alcoholic drinks.

Subjects in control and intervention groups were randomly assigned to six health centers. Therefore, the relationship between the two groups was completely omitted. For this purpose, three pairs of health centers (each pair consisted of 2 centers that were similar in terms of social, economic, cultural, and geographical characteristics) were randomly selected (one pair of centers was located in a city region with higher socioeconomic class, one in the middle socioeconomic class, and one in the higher socioeconomic class).

In each pair of centers, one was assigned to the intervention group and one to the control group. In other words, 3 centers were selected for the control group and 3 for the intervention group. The sample size in each center was chosen to be 28 individuals, and due to the probable loss of 20%, 33 individuals were selected in each center, and the total sample size became 33×6=198. It should be noted that the participants of each center were invited through a public invitation of participating in the research (the inclusion criteria were included in the invitation) which could be found on the clinics’ bulletin. 

The participants were selected using a table of random numbers from women who referred to the clinic. After approval of the Ethics Committee and obtaining informed consent from the participants, all participants were emphatically informed that participation in the study was completely voluntary and they were able to quit at any stage without any restrictions. At the beginning of the study, all of the participants took a pretest.

In the pretest, the participants of both groups filled out the informed consent form, the demographic information questionnaire, and the marital quality scale. The demographic information questionnaire included age, education level, job, spouse age, spouse education level, spouse job, family income level, marriage duration, number of pregnancies, number of children, and addiction background of the couple. Marital quality was measured using the marital quality standard questionnaire. This questionnaire includes 14 questions and aims to evaluate marital quality from different aspects (agreement, satisfaction, and solidarity). It was devised by Busby, Crane, Larson, and Christensen (16). 

This 14-question scale is scored through a 6-point Likert scale ranging from 0 to 5 in a way that score 5 represents completely agree and score 0 indicates completely disagree. The tool consists of three subscales of agreement, satisfaction, and solidarity and a high score indicates higher marital quality indicator. The subscale of agreement consists of 6 items as follows: v. I always agree, iv. I almost always agree, iii. I sometimes agree, ii. I often disagree, i. I almost always disagree, and 0. I always disagree. The subscale of satisfaction includes items 7 to 11 as follows: 0. Always, i. Most of the time, ii. Most often, iii. Sometimes, iv. Hardly ever, and v. Never. The subscale of solidarity consists of items 12 to 14. Item 12 was scored using the following scale: v. Every day, iv. Almost every day, iii. Sometimes, ii. Almost sometimes, i. Hardly ever, and 0. Never, and the items 13 and 14 were scored using scales 0. Never, i. Less than once a month, ii. Once or twice a month, iii. Once or twice a week, iv. Once a day, and v. More. 

In order to obtain the scores related to each dimension, the total scores of questions relevant to that dimension were added up, and to calculate the total score of the questionnaire, the total scores of all questions were added up. Higher scores indicated higher marital quality and vice versa. The tool used in the present study was a standard questionnaire of which validity and reliability were evaluated in previous studies (16). Cronbach’s alpha reliability study of Hollist, Cody and Miller for three subscales of the agreement, satisfaction, and integrity, respectively was reported 79, 80, and 90%. Internal consistency coefficient reliability Cronbach’s alpha of the total questionnaire was 92%. The validity coefficient of 39% was obtained (17).

After the primary assessments, the intervention group was provided with consultation while the control group received no intervention. The target consultation was provided in the form of 8 cognitive behavioral counseling sessions of 90 minutes for 8 weeks (18). Each session involved questions and answers, lecturing, group discussion (in groups of maximum 10 individuals), and presentation of teaching slides. In order to provide the consultation, cognitive therapeutic method was used, and each session included cognitive-behavioral group therapy based on eclectic cognitive-behavioral model (19), and the use of valid sexual resources.

Session 1 and 2 was identifying inefficient beliefs and explaining negative thoughts regarding sexual satisfaction and marital quality, and psychological training was examining the cognitive behavioral model and introducing cognitive distortion regarding sexual dissatisfaction and unfavorable marital quality, homework was revising cognitive distortion.

Session 3 and 4 was examining the homework, and psychological training was examining the methods to fight against cognitive distortion, Homework was practicing identification of cognitive distortion using thoughts recording sheets.

Session 5 and 6 was examining the homework, and psychological training was introducing coping and preventing methods of behaviors and thoughts leading to sexual dissatisfaction, Homework was cognitive reconstructing, completing the sheets of recording thoughts, practicing coping and preventing inappropriate behaviors and thoughts.

Session 7 and 8 was examining the homework, and psychological training was discussing and examining the factors, preventing approaches, returning from sexual dissatisfaction, increasing sexual satisfaction, and improving marital quality, Homework was practicing preventing approaches to deal with return.

The sessions were held in a training class, and the intervention group participants were informed about the date of attending the sessions by phone calls. Moreover, one day before the sessions, the individuals were reminded about the sessions in order to prevent sample loss as much as possible. Consultation was provided by a clinical psychologist. Moreover, the trainers agreed to perform a uniform teaching method.

After the 8th session, both groups took the posttest in which marital quality was evaluated again. It should be noted that after the study, a session about sexual issues was held for the control group, and they were provided with booklets. In order to analyze the collected data, independent samples t test and covariance analysis or change analysis were utilized. All of the tests were carried out at a confidence level of 95%.

## Results

According to the results of the present study, the average age in the control group was 32.58 ± 7.54 years old and in the intervention group 35.04 ± 7.91 years old; so, the two groups were not homogenous in this regard. However, the two groups were homogenous in terms of husbands’ age in the intervention group (37.23 ± 8.01 years old), and in the control group (39.13 ± 7.18 years old), marriage duration in the intervention group (8.95 ± 7.67 years) and control group (8.49 ± 6.99 years), and number of children in the intervention group (1.53 ± 0.96) and control group (1.74 ± 0.86). Moreover, other demographic characteristics such as the level of education in wives and husbands, and drug addiction in wives and husbands, were homogeneous in the two groups (P<0.05) (Table 1).

Covariance analysis was used in order to examine marital quality scores. In this analysis, after modification of the variables of age, marital quality score of agreement and satisfaction before the intervention, as well as income status, marital quality scores were compared between the two groups. Variance analysis and regression coefficients are presented in Table 2. The value of determination coefficient was calculated as 0.85. After the intervention, significant difference was observed between the two groups in all dimensions and total marital quality (P<0.05) and the mean scores of the dimensions increased remarkably (Table 2).

Here, t test was used to compare the two groups before intervention in order to verify if the two groups are different before the intervention, the effect of this difference should be adjusted when comparing them after intervention. 

According to the determined cut-off points based on marital quality scale, all dimensions and total marital quality, a limited number of women had a suitable level of marital quality before the intervention. After the intervention, however, marital quality scores increased, and a significant difference was observed between the two groups regarding all dimensions (P<0.05, Table 3).

**Table 1:** A comparison of demographic characteristics in the two groups


Variable		Control group	Intervention group	P value (%)
		n (%)	n (%)		

Education level	Primary	11 (11.1)	8 (8.1)	0.060
	Secondary	16 (16.2)	10 (10.1)	
	Under diploma	28 (28.3)	26 (26.3)	
	Diploma	35 (35.4)	33 (33.3)	
	University	9 (9.1)	22 (22.2)	
	Spouse education level	Primary	10 (10.1)	3 (7.1)	0.050
Secondary		17 (17.2)	14 (14.1)	
Under diploma		27 (27.3)	19 (19.2)	
Diploma		37 (37.4)	36 (36.4)	
University	8 (8.1)	23 (23.2)	
Spouse addiction	Yes	21 (21.2)	26 (26.3)	0.404
	No	78 (78.8)	73 (73.7)	
Addiction	Yes	1 (1)	1 (1)	1
	No	98 (99)	98 (98)	
Income status	<1,000,000	46 (55.4)	40 (40.4)	<0.001
	>1,000,000	53 (11.1)	59 (59.6)	


**Table 2:** A comparison of the mean scores of different dimensions of marital quality of women before and after the intervention in the two groups


Different dimensions of marital life	Group	Before intervention		After intervention		P value
		Mean + SD	Min-Max	Mean + SD	Min-Max	

Agreement	Control	11.18 + 3.41	3 (19)	11.09 (2.94)	4 (19)	0.603
	Intervention	15.56 + 4.94	3 (28)	25.11 (3.32)	9 (30)	<0.001

						Covariance analysis with adjustment the effect of age, income, agreement score before intervention
P (Independent t test)	<0.001		<0.001	
Satisfaction	Control	7.60 (1)	2.57 (7.46)	17 (1)	3.04 (7.60)	0.489
	Intervention	9.89 (8)	2.13 (16.53)	18 (1)	4.35 (9.89)	<0.001

						Covariance analysis with adjustment the effect of age, income, satisfaction score before intervention
P (Independent t test)	<0.001		<0.001	
Solidarity	Control	7.64 (2.73)	1 (14)	7.49 (2.96)	1 (14)	0.382
	Intervention	8.1 (2.89)	1 (16)	15.90 (2.54)	9 (20)	<0.001

						Covariance analysis with adjustment the effect of age, income
P (Independent t test)	<0.001		0.199	
Total	Control	26.41 (6.37)	9 (45)	26.05 (5.99)	9 (64)	0.261
	Intervention	33.60 (9.93)	6 (67)	57.54 (5.94)	34 (69)	<0.001

						Covariance analysis with adjustment the effect of age, income, total score before intervention
P (Independent t test)	<0.001		<0.001	


**Table 3:** Frequency distribution of suitable marital quality based on cut-off point before and after the intervention in both groups


Dimensions of married life	Before intervention n (%)		Comparison beforeintervention^* ^ (P value)	After intervention n (%)		Comparison afterintervention^** ^ (P value)
	Control group	Intervention group		Control group	Intervention group	

Agreement	0 (0)	14 (100)	<0.001	0 (0)	88 (100)	<0.001
Satisfaction	1 (4.8)	20 (95.2)	<0.001	2 (2.2)	91 (97.8)	<0.001
Solidarity	16 (42.1)	22 (57.9)	0.279	15 (13.4)	97 (86.6)	<0.001
Total	0 (0)	7 (14.3)	0.014	0 (0)	94 (98)	<0.001


*; For comparing agreements, satisfaction and solidarity between the two groups before the intervention, chi-square test was used and to compare total marital quality, Fisher’s exact test was used and **; For comparing solidarity between the two groups after the intervention, Chi square test was used of and to compare other cases (because the two groups at baseline were not similar), Wald test resulting from logistic regression with adjustment for the effect of the intervention, was used.

## Discussion

The present study was conducted in order to examine the effect of cognitive behavioral consultation on marital quality among women. The results indicated that cognitive behavioral consultation led to an increase in total marital quality and all its dimensions (solidarity, satisfaction, and agreement). The individuals had a low marital quality before the intervention; however, after the intervention, the scores rose remarkably showing the efficacy of consultation in improving marital relationships. 

This means that cognitive behavioral consultation concerning sexual issues, led to an increase in the total score and dimensions of marital quality in the intervention group. Previously, Young and Carlson (20) showed that cognitive behavioral marital consultation can affect the quality of the individuals’ marriage, which is in line with the present study.

In the present study, during the sessions, the wives were taught to solve their sexual and marital problems with the help of their husbands. When the problem is regarded as a joint issue, a single individual is not considered as the cause, and the couples become aware of their roles in the emergence of the problem, so, they stop blaming one another, and there will be less argument between them.

Numerous indices are used to show marital quality. Perry (21) proposed satisfaction with marriage, spending time together, management of conflicts, prediction of divorce possibility, and frequency of conflicts between the couples as marital quality indices.

Satisfaction with sexual relationships is an important factor in marital relationships. Individuals who are highly satisfied with the sexual relationship they have with their spouses, have a remarkably higher quality of life, express higher love and interest to their spouse, and have higher levels of agreement, solidarity, and satisfaction in their marital relationships (22). In this regard, Khajeh et al. (23) and Mangeli (24) indicated the positive effect of sexual counseling on improving marital relationships and satisfaction.

Regarding marital satisfaction and improvement of marital quality in dimensions of solidarity and agreement, the wife’s and husband’s understanding of one another’s behavior is significantly important. Cognitive behavioral therapy tries to fix the incorrect attitude toward spouse and wrong myths about marital relationships and create skills to establish more effective communication and problem solving (25).

In a study, Akbarzadeh (26) indicated that cognitive behavioral education of the couples was effective in family performance and subscales of problem solving, communication, emotional companionship, emotional involvement, and behavior control among divorced applicants. Cognitive foundations of cognitive behavioral therapy of couples highlight the couples’ understanding of one another and consider understanding as an inseparable part of change in couples. Finally, the philosophical foundation of this understanding is that change in behavior alone is not enough to correct inefficient interactions and there should be an emphasis on how individuals think about their relationships and their incompatible behavioral patterns (27).

In the study of Khanjani Veshki et al. (28), carried out on 30 women referring to counseling centers in Qom, Iran, researchers utilized a researcher-designed marital quality questionnaire and 6 sessions of sex education. They showed that the intervention could improve marital quality and its dimensions. Due to the role of sexual relations in consolidating marital relationships and its quality, marital consultation leads to an increase in sexual satisfaction, and the women participating in the educational programs reach higher sexual pleasure and express more passion and affection in their marital relationships. 

Moreover, Salimi and Fatehizadeh (29) showed that sex education by cognitive behavioral method enhances the married women’s knowledge, self-expression, and sexual intimacy, which is in line with the results of the present study.

With regard to the significance of our results, it can be stated that the cognitive behavioral sex consultation used in the present study could improve all dimensions of marital relationships by emphasizing on cognitive dimensions of sex consultation such as pinpointing and challenging the common sexual wrong beliefs through group discussion, determination and improvement of individuals attitude toward sexual activity, presentation of realities and importance of satisfying sexual desires, the role of sexual relation in general relationships and quality dimensions of marital relationships.

In the cultural context of Iranian society, women and men have poor and inaccurate sexual knowledge, and do not have access to reliable sources in this regard. Moreover, they have a lot of incompatible and illogical thoughts, beliefs, attitudes, and understanding of sexual issues which affect the couples’ sexual relation. In cognitive behavioral approach, attention is paid to sex education as Iranian couples had not obtained this knowledge by a reliable method (30, 31). According to the mentioned issues, it is determined that in the Iranian culture, paying attention to cognitive factors is highly important to resolve sexual problems, dysfunction, and dissatisfaction, and neglecting them leads to a decrease in treatment success. 

Cognitive behavioral family therapy training equip the couples with skills which are necessary for marital life, and can be generalized to other levels of marital and social levels of life. According to the Iranian culture and since an extensive range of treatment techniques and methods is used, cognitive behavioral method can be effectively employed as an extensive method to treat behavioral problems and positively change couples. Using a large population size from various clinics of the city was the strength of the present study, and failure to follow them after the study was one of its limitations.

Due to the effectiveness of the method used in the present study, it is suggested that techniques of this approach could be utilized by family and marriage counselors to increase sexual satisfaction, solve marital conflicts and improve marital quality of the couples’ life. Moreover, the applicable drills and skills of this method in the form of educational sessions, workshop, videos, and pamphlets should be employed to prevent marital problems.

## Conclusion

Due to the role of sexual relations in consolidating marital life, cognitive behavioral consultation as an effective method for improving marital quality, especially after an agreement, can be employed in health care centers to improve the couples’ relationship and reduce divorce rates.
